# A Psychometric Study of the Perinatal Assessment of Maternal Affectivity (PAMA) for the Screening of Perinatal Affective Disorders in Mothers

**DOI:** 10.3390/healthcare11060907

**Published:** 2023-03-21

**Authors:** Franco Baldoni, Francesca Agostini, Grazia Terrone, Giulia Casu, Michele Giannotti

**Affiliations:** 1 Department of Psychology, University of Bologna, 40127 Bologna, Italy; 2 Department of History, Cultural Heritage, Education and Society, University of Rome Tor Vergata, 00133 Rome, Italy; 3 Department of Psychology and Cognitive Sciences, University of Trento, 38068 Trento, Italy

**Keywords:** perinatal, affective disorders, depression, anxiety, mothers, fathers, PAPA, PAMA, screening

## Abstract

Recently, empirical evidence from perinatal studies has led researchers to pay more attention to fathers. The need to evaluate male suffering led at first to using the same screening tools developed for mothers. However, these instruments present validity concerns with fathers, and today the need to assume a gender-based perspective is clear. The Perinatal Assessment of Paternal Affectivity (PAPA) is a self-reported questionnaire for the screening of a variety of psychological and behavioral dimensions related to affectivity as experienced by fathers during the perinatal period. In the present study, the psychometric properties of the maternal version of the scale (Perinatal Assessment of Maternal Affectivity; PAMA) were examined. The study, based on 225 mothers and their partners (*n* = 215), used a cross-sectional design with a single assessment at the third trimester of pregnancy. Results indicated a one-factor structure for a seven-item version of the PAMA, which showed adequate internal consistency reliability and was associated in the expected direction with other clinically relevant variables (depression, psychological distress, perceived stress and dyadic adjustment). The findings suggest the usefulness of developing gender sensitive screening tools for the detection of perinatal affective disorders.

## 1. Introduction

For decades, research on perinatal affective disorders (particularly postpartum depression) has focused almost exclusively on mothers. The role of the father has been often underestimated by health professionals (pediatricians, gynecologists, obstetricians and nurses), since pregnancy and childbirth have been traditionally considered as predominantly female issues. This attitude, called “maternal gatekeeping” [1,2], may increase the exclusion of fathers, legitimizing paternal disengagement in childcare across the transition to parenthood [3]. Furthermore, fathers are often reluctant to ask for help and to communicate their psychological and behavioral difficulties. Men also tend to show their emotional difficulties in a different way than women, minimizing the depressive aspects and the need for help. Consequently, paternal affective disorders tend to be considered less severe and are clinically underreported. On this matter, it is relevant to note that self-reported tools designed for the evaluation of female depressive symptoms are usually used for screening and diagnosis in males, thus leading to a potential underestimation of paternal suffering [4]. These scales may not be sensitive in capturing the complex clinical picture of paternal perinatal psychological distress.

For instance, the Edinburgh Postnatal Depression Scale (EPDS), initially in 13-item [5] and then 10-item versions [6], is the most popular tool for the screening of perinatal affective disorders in mothers and fathers and assesses depression and anxiety. It was validated for the first time in Anglo-Saxon mothers [6] and later in mothers of several other countries (e.g., Italy, Sweden, Spain, Portugal, France, the Netherlands, Australia, Saudi Arabia, India, China and Japan). The first attempt to validate the EPDS on fathers, conducted on an Australian sample, identified a cut-off score to detect symptoms related to depression or anxiety disorders significantly lower (five/six) than the one used for mothers (seven/eight) [7]. This difference has been confirmed by a study on a sample of 192 British fathers [8] that compared the EPDS scores with the results of structured clinical interviews. The questionnaire proved to be a valuable screening tool for the detection of at-risk fathers, but, in these cases, a cut-off score > 10 was recommended for the assessment of depression and >8 for generalized anxiety disorder symptomatology. A recent systematic review and meta-analysis [9], including seven studies and a total of 2393 fathers, confirmed that the EPDS has acceptable properties for detecting paternal postpartum depressive symptomatology with cut-off scores ranging from seven to ten to optimize the balance between sensitivity and specificity, therefore decreasing the risk of false positives. However, as highlighted by Matthey [10], only a small number of studies on fathers followed these guidelines and the respective cut-off scores. Therefore, prior findings need to be interpreted with caution, considering the heterogeneity of study samples in terms of education, cultural and socio-economic background and time of EPDS administration.

Moreover, previous research on perinatal depression based on self-reported questionnaires pointed out other inconsistent results. A study conducted in Japan [11] evaluating a sample of 146 fathers at the fourth week post-partum, and administering both the CES-D (cut-off ≥ 16) and the EPDS (cut-off ≥ 9), showed significant differences in terms of depression rates using the CES-D and the EPDS (7.5% and 11.6%, respectively). The same difference emerged in mothers, with rates of depression of 24.2% (CES-D) and 28.1% (EPDS), respectively. In a Danish study [12], 5% of fathers assessed using the EPDS were identified as at-risk of postnatal depression; using the Gotland Male Depression Scale (GMDS) [13] (a specific questionnaire for depression in males) on the same sample, the at-risk rate dropped at 3.4%.

Despite the methodological challenges, meta-analyses and systematic reviews [14,15,16,17] have shown that across the perinatal period fathers manifest affective disorders with a frequency almost comparable to that of mothers. Meta-analyses [14,16] revealed that the rate of perinatal depression in fathers ranges from 8.4% to 10.4% (compared with 13% in women). Therefore, new fathers may experience depressive symptoms with a frequency that is almost three times greater than that of the general population [18]. Furthermore, previous research has shown the interrelationship of maternal and paternal perinatal depression, suggesting the need to consider a dyadic framework [14,19].

Unfortunately, no reference is made to paternal perinatal depression in the DSM-5 [20] (American Psychiatric Association, 2013), notwithstanding the high prevalence rates of Paternal Perinatal Depression (PPND) that confirm the urgency to implement specific routine screening for identifying early signs of depression in fathers [21,22,23]. In this regard, it is essential to consider that the clinical expression of depressive symptoms in fathers is milder and less defined than in mothers. In fact, perinatal psychological distress in fathers could be manifested trough “externalizing” strategies such as drinking alcohol, smoking or drug abuse, behavioral acting outs and loss of impulse control, especially anger attacks [24,25,26]. Specifically, depressive symptoms in fathers can be often accompanied by other affective and behavioral disorders [4] such as:

(1) anxiety disorders (generalized anxiety disorder, panic attacks, phobias, post-traumatic stress disorder, obsessive–compulsive disorders), affecting up to 18% of fathers [15];
(2) abnormal illness behavior (somatization disorders, functional medical syndromes, hypochondriacal concerns) [27,28];
(3) behavioral acting out and externalizing disorders (anger attacks, violent or dangerous conduct, compulsive physical or sexual activity, extramarital affairs, running away from home or at work, relationship conflicts) [28,29];
(4) addictions (smoking, alcohol, drugs) and other addictive disorders (such as those from gambling, social media or the internet) [23,29].

These disorders, which can overlap or mask depressive symptoms, tend to occur more frequently in the third trimester of pregnancy and in the first three to four months after childbirth. For all these reasons, the term Paternal Perinatal Affective Disorder (PPAD) [4] has been proposed to replace the term Paternal Perinatal Depression, considering the broader spectrum of symptoms that may occur in fathers during the transition to parenthood.

Therefore, the screening of perinatal affective disorders requires gender sensitive tools, including the assessment of several signs and symptoms of psychological distress. However, the most common and usual screening tools in this field are developed considering female over male signs and symptoms and not vice versa. Only in recent years have the growing concerns on paternal mental health during the perinatal period [4] pushed some researchers to develop different and specific screening and assessment tools for fathers, such as the Dads Questionnaire [30] and the Perinatal Assessment of Paternal Affectivity (PAPA) [23]. In a recent cross-sectional study with a 3 month test–retest involving 385 (T1) and a sub-sample of 111 (T2) fathers [23], respectively, the PAPA showed adequate internal consistency and test–retest reliability. A single factor common to the male disorder was evidenced by confirmatory factor analysis. Validity was also supported by significant correlations between PAPA and other clinically relevant variables: PAPA scores correlated positively with measures of depressive symptoms, psychological distress and perceived stress, with medium to large effect sizes, and negatively and moderately with dyadic adjustment [23]. Italian validation data provided initial evidence of validity and reliability of the PAPA as a simple screening tool to detect affective disorder symptomatology in fathers during the perinatal period.

However, little is known about the reliability and validity of the same tool in mothers. This could be of particular interest for the screening of different psychological dimensions in mothers during the transition to parenthood. Therefore, a maternal version of PAPA was developed called Perinatal Assessment of Maternal Affectivity (PAMA). The PAMA includes both clinically relevant and poorly investigated signs and symptoms that have been related to maternal depression, such as interpersonal problems and somatization. Specifically, previous studies found that women may exhibit a high level of somatization, suggesting that it may play a critical role in identifying maternal depression, particularly during the prenatal period [31,32]. Similarly, interpersonal problems in mothers during pregnancy, such as marital dissatisfaction, are still poorly understood, although they have been linked to poor perinatal mental health in both parents [33,34]. Thus, a more comprehensive assessment including several psychological dimensions may improve maternal depression screening practices in clinical settings.

Therefore, in this study, we aimed to examine the psychometric properties of the (PAMA) in terms of internal structure, internal consistency reliability and relations with other variables. We hypothesized that PAMA would show adequate reliability and validity. For evidence of validity based on internal structure, we expected that the same one-factor model of the PAPA would be confirmed for the maternal version (PAMA). For evidence of validity based on relations with other clinical variables, we expected that the PAMA would correlate positively with depression, psychological distress and perceived stress scores, and negatively with dyadic adjustment. In addition, we compared maternal and paternal scores and explored correlations among PAMA and PAPA scores. Based on previous evidence [33,35], we expected higher perinatal distress scores in mothers than in fathers, and hypothesized a significant positive correlation between mothers’ and fathers’ reports of perinatal distress.

## 2. Materials and Methods

### 2.1. Study Design and Participants

The current study used a cross-sectional design with a single assessment of mothers and their partners at the third trimester of pregnancy.

The participants were recruited from three Italian perinatal health services between January 2017 and September 2019 during regular gynecological visits. Inclusion criteria were: age ≥ 18 years, being in the third trimester of pregnancy and Italian speakers. Maternal and/or fetal health problems, pregnancy complications (e.g., abnormal placenta position or poor fetal growth), threatened preterm labor and current psychiatric diagnoses were defined as the exclusion criteria of the study. Participation in the study was voluntary. Clinical psychologists proposed the research to the parents, who could withdraw from the study at any stage of the research. All the mothers who were invited agreed to participate. This study obtained ethical approval from the regional Institutional Review Board (CEIIAV, n. 1607).

### 2.2. Measures

#### 2.2.1. Socio-Demographic Data

Socio-demographic information (including age, nationality, region of residence, education, occupation, marital status and number of children) and the presence of stressful life events in the previous six months such (e.g., job loss, divorce and mourning) were collected through questionnaires.

#### 2.2.2. Perinatal Assessment of Paternal/Maternal Affectivity (PAPA/PAMA)

PAPA and PAMA [23,36,37,38] are new self-reported questionnaires recently proposed as a screening measure to provide a global score of perinatal distress, consistent with the comprehensive definition of PPAD. PAPA and PAMA were originally developed by a group of experts in perinatal psychopathology who first autonomously proposed a pool of items. Both were originally developed in English and then translated back into Italian. These screening tools are designed to identify parents at risk of developing a perinatal affective disorder.

Specifically, in contrast with traditional screening tools, the PAPA has been developed considering the wide array of signs and symptoms manifested by fathers during the perinatal period and consists of 8 items assessing anxiety, depression, perceived stress, irritability/anger, relationship problems (including couple, family, friends and at work), abnormal illness behavior (somatization, functional medical syndromes and hypochondriac complaints), physiological problems (sleeping, eating or sexual desire), addictions (smoking, drinking alcohol, taking drugs, gambling and compulsive use of the Internet) and other risky behaviors (such as driving at high speed, dangerous sports or taking unnecessary risks at work). Fathers are asked to rate the severity of their symptoms and behaviors in the last 2 weeks using a four-point Likert-type scale (0 = Not at all, 1 = A bit, 2 = Moderately, 3 = A lot). The first Italian validation of the paternal version (PAPA) revealed adequate psychometric properties [23].

The Perinatal Assessment of Maternal Affectivity (PAMA) is the maternal version of PAPA, with items written in the feminine form, as the aim of the PAMA is the global assessment of maternal affectivity during the perinatal period. PAMA items are displayed in Table 1.

The PAPA and the PAMA are available in 3 versions: Standard (for assessment throughout the perinatal period), Prenatal (for assessment in the prenatal period only) and Postnatal (for assessment in the postnatal period only). In this study, we used the prenatal version of the questionnaires.

#### 2.2.3. Depressive Symptomatology

We used the Center for Epidemiologic Studies Depression Scale (CES-D) [39], to assess depressive symptoms in mothers and fathers. The CES-D is a self-reported measure with 20 items rated on a four-point Likert-type scale (0 = never to 3 = always). Participants responded according to how often they perceived listed symptoms in the past week. The questionnaire showed good psychometric properties [40], and it is one of the most widely used scales to assess depression in both parents across the transition to parenthood [41]. In the current study, we used the Italian version of the scale [42], which showed good internal consistency in this sample (*α* = 0.81).

#### 2.2.4. Psychopathological Symptoms

The Symptom Checklist-90-Revised (SCL-90-R) [43] is a 90-item self-reported questionnaire to assess several dimensions of psychological distress and symptoms of psychopathology. Participants are asked to rate their symptoms’ severity during the past week using a five-point Likert-type scale (0 = not at all to 4 = extremely). The questionnaire yields to 9 subscales (Obsessive–Compulsive, Interpersonal Sensitivity, Depression, Anxiety, Hostility, Phobic Anxiety, Paranoid Ideation and Psychoticism) and 3 general scores (Global Severity Index, GSI; Positive Symptom Distress Index; and Positive Symptom Total). In this study, we considered GSI score, as it represents a global index of the intensity of distress perceived by the subject. We used the Italian validated version [44], which has shown adequate psychometric properties. Internal consistency in the present study was *α* = 0.96.

#### 2.2.5. Perceived Stress

The Perceived Stress Scale (PSS) [45] is a 10-item self-reported measure to assess feelings and thoughts related to stress during the last month. Respondents are asked to rate their level of stress on a five-point Likert scale (from 0 = never to 4 = very often). This scale showed adequate reliability and construct validity [45], and has been used in the context of perinatal research [46]. The Italian version of the PSS [47] was used in the current study. Reliability estimate in this study was *α* = 0.75.

#### 2.2.6. Dyadic Adjustment

To assess the quality of couple relationships, we used the Dyadic Adjustment Scale (DAS) [48], a self-reported questionnaire of 32 items assessing relationship quality in married or cohabiting couples. The questionnaire includes 4 subscales, namely dyadic satisfaction, dyadic cohesion, dyadic consensus and affectional expression. Participants were asked to rate each item on a five- or six-point scale (e.g., 0 = always disagree to 5 = always agree). Two items have a dichotomous answer (Yes = 0, No = 1). A DAS total score was used in the present study by summing all items, with higher scores indicating higher relationship quality. We used the Italian version of the DAS [49], which has been previously used to examine couple adjustment during the transition to parenthood [50]. Internal consistency reliability in this study was *α* = 0.87.

### 2.3. Data Analysis

To test the hypothesized one-factor model of the PAMA, we applied confirmatory factor analysis (CFA) using the robust weighted least square with mean and variance adjustment (WLSMV) estimation method, which is appropriate for ordinal data [51]. Model fit was considered adequate if the root mean square error of approximation (RMSEA) was ≤0.08, the comparative fit index (CFI) and the Tucker–Lewis index (TLI) were ≥0.95 [52] and the weighted root mean square residual (WRMR) was ≤1.0 [53]. In case of poor fit, we inspected modification indices and expected parameter change to identify areas of misfit and re-specify the model [54]. Model comparison was performed with the adjusted *χ*^2^ difference test (Δ*χ*^2^) using the Mplus version 7 DIFFTEST function [55]. A significant adjusted Δ*χ*^2^ would indicate improvement in model fit. The best-fitting model was selected and used in subsequent psychometric analyses.

Internal consistency reliability was assessed using McDonald’s *ω* and Cronbach’s *α*, with values > 0.70 indicating adequate reliability [56,57,58], and corrected item-total correlations, with values > 0.30 considered acceptable [58].

To collect evidence of validity based on relations with other variables, we computed Spearman’s correlation coefficients between PAMA and CES–D, PSS, GSI and DAS total scores. We also examined differences in PAMA scores between groups based on number of children (None vs. One or more), nationality (Italian vs. Other) and stressful life events (None vs. One or more) using Mann–Whitney *U* tests.

Differences between partners in perinatal affective symptomatology scores were tested using Wilcoxon signed-rank test in a subsample of mothers whose partners completed the PAPA (*n* = 215). Spearman’s correlations between fathers’ and mothers’ scores were also computed.

Sample size was determined a priori as to have 5-to-10 observations for each estimated parameter in the CFA model [59]. Statistical significance was set at *p* ≤ 0.05. Effect size interpretation was based on *ρ* (correlation analyses) and *r* (Wilcoxon signed-rank tests), with values of 0.10 considered small, 0.30 medium and 0.50 large [60], and *ε*^2^ (Mann–Whitney *U* tests) of 0.04 considered small, 0.25 medium and 0.64 large [61].

## 3. Results

### 3.1. Participants’ Characteristics

The total sample of this study consisted of 225 mothers (mean age = 31.95 years, SD = 4.99, range 19–46 years) and their partners (*n* = 215, mean age = 35.30 years, SD = 6.56, range 21–58 years), assessed at the third trimester of pregnancy. Most of the participants (197 mothers, 161 fathers; 87.5% and 74.8%, respectively) were Italian. Ninety-one mothers (40.4%) and 108 fathers (50.2%) were primiparous. Among the study participants, 66 mothers (29.3%) and 58 fathers (27%) reported to have experienced one or more stressful life events in the previous six months. Stressful life events that were reported most frequently by mothers (*n* = 66, 29.3%) were mourning or illness of a close relative/friend (*n* = 16, 24.2%), family conflicts (*n* = 8, 12%), illness (*n* = 8, 12%) and marriage (*n* = 8, 12%). For fathers who reported stressful live events (*n* = 52, 24.2%), the most frequent were illness or loss of a close relatives/friends (*n* = 16; 30.8%), family conflicts (*n* = 10; 19.2%), serious work-related problems (*n* = 9, 17.3%) and romantic disappointments (*n* = 5, 9.6%).

Descriptive statistics of mothers and fathers are displayed in Table 2.

### 3.2. Internal Structure of PAMA

**Figure 1:**
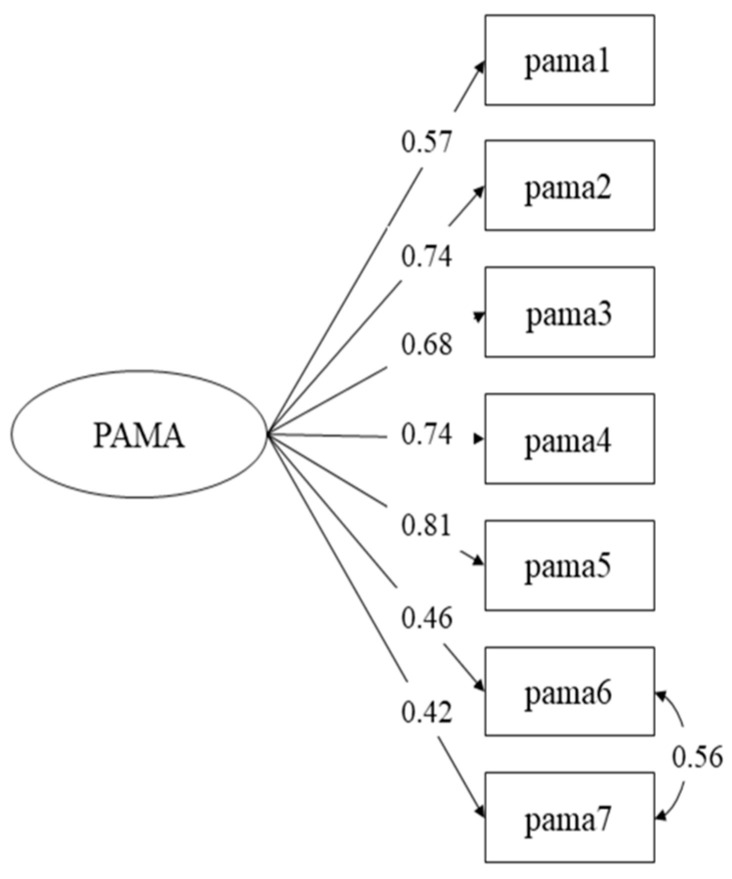
PAMA measurement model with standardized parameters.

The fit of the one-factor model hypothesized for the PAMA was not satisfactory, *χ*^2^_20_ = 101.178, *p* < 0.001, RMSEA = 0.134, CFI = 0.894, TLI = 0.851 and WRMR = 1.224. Examination of factor loadings indicated that the loading of item eight (which refers to behavioral disorders and addictions) was not significant (standardized estimate = 0.211, *p* = 0.059); therefore, this item was removed. The one-factor model was re-estimated with seven items, showing unacceptable fit, *χ*^2^_14_ = 95.512, *p* < 0.001, RMSEA = 0.161, CFI = 0.897, TLI = 0.845 and WRMR = 1.303. Inspection of modification indices suggested that estimating the residual correlation between item six and item seven would decrease the *χ*^2^ by 70.338, with an expected parameter change of 0.759. Thus, we re-specified the model by including this correlation. The fit of the re-specified model was adequate according to all fit indices, *χ*^2^_13_ = 26.621, *p* = 0.014, RMSEA = 0.068, CFI = 0.983, TLI = 0.972 and WRMR = 0.645, and the Δ*χ*^2^ test indicated that improvement in model fit was statistically significant (Δ*χ*^2^_1_ = 41.281, *p* < 0.001). This model was thus selected for the PAMA, which includes seven items. Standardized factor loadings ranged between 0.42 and 0.81 (*p* < 0.001). Figure 1 shows the measurement model of the final seven-item PAMA, which we refer to hereafter as PAMA.

### 3.3. Internal Consistency Reliability

McDonald’s *ω* was 0.76, Cronbach’s *α* was 0.78 and corrected item-total correlations ranged from 0.42 to 0.60, indicating adequate internal consistency reliability for the PAMA. When item eight was also considered, internal consistency was still acceptable based on McDonald’s *ω* and Cronbach’s *α*, which were both equal to 0.76. However, the corrected item-total correlation of this item was only 0.13, further supporting its removal.

### 3.4. Relations with Other Variables

PAMA scores correlated significantly (*p* < 0.001) and positively with the criterion measures CES-D (*ρ* = 0.54), GSI (*ρ* = 0.61) and PSS (*ρ* = 0.57), with large effect sizes; additionally, PAMA significantly and negatively correlated with the DAS (*ρ* = −0.39), with a medium effect size.

Mann–Whitney *U* tests indicated no differences in PAMA scores between groups based on number of children (*U* = 5267.00, *z* = −1.30, *p* = 0.19) or presence of stressful life events (*U* = 4514.50, *z* = −1.65, *p* = 0.10). There were significant differences between groups based on nationality, with Italian mothers reporting significantly higher PAMA scores than foreign mothers, with a small effect size (*U* = 1888.50, *z* = −2.45, *p* = 0.01, *ε²* = 0.03) (Table 3).

### 3.5. Relation between PAMA and PAPA Items

Descriptive statistics and tests of differences between mothers’ and fathers’ scores are displayed in Table 4.

When considering individual items, mothers scored slightly higher than fathers in anxiety, depression and anger items, and moderately higher than fathers in somatization and sleep/eating/sexual desire items. In the addiction/risky behavior item, fathers scored slightly higher than their partners.

Considering mean scores across all items, PAMA scores were significantly moderately higher than PAPA scores.

Correlation analysis indicated a significant and moderate positive correlation between the PAMA and PAPA mean scores (*ρ* = 0.37, *p* < 0.001).

## 4. Discussion

The PAPA and PAMA have been specifically developed for the screening of affective disorders in parents during the perinatal period.

A first recent publication [23] showed promising psychometric properties of the PAPA regarding its use in the perinatal period for the detection of fathers’ symptomatology. The present study aimed to explore the psychometric characteristics of the PAMA, which was administered to expectant mothers at the third trimester of pregnancy.

Altogether, the findings showed adequate psychometric characteristics for a seven-item version of the PAMA in terms of internal consistency reliability and validity based on internal structure and relations with other clinically relevant variables.

To collect evidence of validity based on internal structure, we performed CFAs and tested whether the same one-factor model of the male version of the tool, the PAPA, held for the PAMA. Compared with the PAPA, an item (item eight) was discarded from the PAMA based on CFA results. Indeed, the male version of the questionnaire was originally developed as a specific tool for screening fathers’ perinatal symptomatology. Theoretical and clinical framework for the PAPA was based on and supported by recent scientific literature [4,9,62,63] emphasizing the need to assess the presence and intensity of different psychopathological dimensions. For this reason, its eight items reflect several affective symptoms (not just depressive and anxiety symptoms but also addictive and risky behaviors, etc.) that characterize paternal affective suffering differently from maternal experiences. The PAMA, on the other hand, was developed to evaluate the same items in mothers, assuming a greater specificity of the PAPA for fathers. Indeed, women, compared with men, tend to manifest perinatal affective disorders differently, in particular through ‘typical’ depressive and anxious symptomatology, rather than through externalizing disorders such as acting out, anger attacks or addictions [28,64]. Based on this, the nonsignificant factor loading of item eight (“I have felt the need to smoke, drink alcohol, use drugs, gamble or use the internet more than usual; or felt the need to take risks, e.g., driving very fast, doing dangerous sports, unnecessary risks at work, etc.”) found for the PAMA in the present study is not surprising. This indicates that addictive and risky behaviors are not included in the conceptualization of maternal affectivity during the perinatal period, and thus seem to be more specific for ‘possibly depressed’ fathers rather than for mothers.

Nevertheless, it is relevant to note that the one-factor model of the PAMA includes different psychological dimensions, such as somatization and interpersonal problems, that are generally excluded from standard screening measures for perinatal depression. This suggests that PAMA may provide an effective and global screening addressing different clinical manifestations that are usually poorly explored in the regular follow up in the perinatal period. The application of PAMA in clinical settings might therefore improve the identification of clinically relevant signs and symptoms of maternal depression since pregnancy, allowing early interventions for at-risk mothers.

Further analyses were performed to collect evidence of validity based on relationships with other variables. The PAMA showed significant correlations, which were in the expected directions, with relevant criterion measures. Specifically, PAMA scores strongly and positively correlated with scores on measures of depression, perceived stress and global severity of psychological symptoms. These results constitute an evidence of validity for the PAMA as they highlight, first of all, the association between a measure on perinatal symptomatology and a very well-known depression measure, the CES-D, as frequently reported by the literature [65]. Another interesting result was the association between higher perinatal maternal symptomatology and higher levels of perceived stress, which is consistent with the findings of previous studies [66,67]. Additionally, a significant association was found between PAMA scores and the GSI of SCL-90-R, in line with previous studies documenting strong correlations between perinatal distress (including PAPA) and general psychopathological symptoms in expectant parents [23,68].

The PAMA were negatively associated with DAS scores, in line with the relationship between higher perinatal depression and a lower quality of marital relationship and dyadic adjustment found in other studies [69,70].

PAMA scores were also analyzed in relation to socio-demographic variables. Significant differences were found in PAMA scores according to nationality, with Italian mothers reporting higher scores compared with mothers of other nationalities. Nonetheless, it is of note that the number of non-Italian mothers was limited; thus, these results should be interpreted with caution and require further replication with wider representative samples. No differences emerged regarding the relationship between PAMA and parity nor the occurrence of stressful events. Regarding the number of children, the parenting literature shows a mixed picture, with some studies supporting a relationship between maternal depression and multiparity [71], and others underlining a negative influence of primiparity [72]; of course, the significant role played by already having other children or not has to be considered within the socio-cultural and economic background of the study sample. With respect to the occurrence of recent stressful events, a significant relationship between maternal depressive symptomatology and the presence of stressful life events has been reported in previous literature [73,74]; however, due to different instruments and categories of stressful events used across studies, and the different characteristics of the study samples, this relationship could not be found systematically. Globally, these findings would benefit from a further deepening.

We also examined the relationship between PAMA and PAPA. When considering mean scores across all items, the results showed that PAMA scores were higher than PAPA scores; this result is in line with most of the international literature, emphasizing that depressive symptomatology tends to occur more frequently and more severely in mothers compared with fathers during both the prenatal and the postnatal period [4,16,75]. However, there is enough evidence from the literature to legitimate paternal perinatal depression as a clinical entity, therefore deserving special attention regarding its identification and treatment [76]. When considering individual items, mothers scored higher than fathers in anxiety, depression and anger items with a small effect size, and in somatization and sleep/eating/sexual desire items with a medium effect size.

Pregnancy is a time of intense physical and psychological change that increases a woman’s vulnerability to anxiety, depression and anger [77]. The expression of perinatal psychological distress in men can be displayed through a wide array of clinical manifestations. Male depressive symptoms are generally milder and less defined than those in mothers [4].

Although the final PAMA does not included item eight, which refers to addiction and risky behaviors, we nonetheless compared scores across mothers and fathers for the sake of completeness. Fathers scored slightly higher than mothers in this item. We have to remember that the clinical expressions of paternal perinatal depression are different from those observed in maternal perinatal depression, since men often exhibit externalizing symptoms defined as depressive equivalents to hide their depression condition [78].

Finally, PAMA and PAPA total scores were significantly and positively correlated with a medium effect size, meaning that as mothers reported higher levels of perinatal distress, so did fathers. This finding reflects what already emerged from previous studies reporting significant correlations between mothers’ and fathers’ affective states and depressive symptoms across the perinatal period [35,79]. Recent systematic reviews and meta-analyses [80,81] pointed out how mental health states in mothers and fathers influence each other during the whole perinatal period, emphasizing the relevance of considering the care of parents within a systemic perspective.

The results of this study seem to confirm that PAMA for mothers, and PAPA for fathers, can be considered a useful tool for the screening of perinatal affective disorders in mothers. It is important to underline that PAMA cannot be considered as a ‘gold standard’ for screening mothers, because it was not developed for this specific use, unlike other valid and reliable tools, such as the EPDS for depression. In any case, it is useful to remember that most of these tools were developed based on the symptomatology of mothers and are less valid for fathers.

This study has some limitations that need to be overcome by further research. First, a larger sample of mothers and fathers could make results more statistically relevant; additionally, our sample was composed almost exclusively of Italian couples, which limits the generalizability of the findings; it is recommended to develop further research on parents from other cultural backgrounds, languages and countries.

Second, we did not calculate any cut-off score to differentiate data from parents diagnosed with perinatal affective disorder from those without diagnosis. The identification of an appropriate cut-off score for PAPA/PAMA will be the subject of future studies by our research group. In any case, it is useful to note that the calculation of cut-off scores for this kind of screening questionnaires might be useless or highly open to criticism due to methodological, linguistic and socio-cultural issues [10]. In fact, searching appropriate cut-off points for this kind of tool requires the use of a more refined diagnostic methodology (semi-structured, psychiatric interviews) and a careful consideration of methodological, cultural and linguistic factors that might influence the results.

Third, this is a cross-sectional study, as the sample was assessed only at the third trimester of pregnancy. We did not consider the study of fathers’ and mothers’ symptomatology over time, but we are aware that trajectories of perinatal affective disorders should be evaluated going from the prenatal to the postnatal period, particularly the second trimester, during which paternal affective disorders frequently tend to occur [3,14,16]. More longitudinal research on perinatal affective disorders is needed to clarify if mothers and fathers show different patterns of affective disorders across the perinatal period.

Lastly, the use of several criterion tests was necessary because PAPA and PAMA evaluate several psychological dimensions (depression, anxiety, relational problems, behavioral disorders, addiction, etc.), which no single screening questionnaire evaluates. The use of different questionnaires necessarily made the study and the statistical evaluation more complex and possibly subject to methodological drawbacks.

## 5. Conclusions

The PAMA, adapted for mothers, has shown adequate psychometric characteristics in this study, which suggests that it can be considered a useful tool for the screening of perinatal maternal affective disorders. Its use would be especially suitable in busy healthcare settings, as it is very short and not time-consuming and easy to administer and interpret, minimizing patient and staff burden. However, the PAMA cannot be considered a reference for the perinatal screening of mothers, as well-known, valid and reliable screening questionnaires of mothers’ perinatal symptomatology already exist, such as the EPDS for depression. The PAMA was in fact developed along with the PAPA in a context of research and clinical exploration of affective disorders which characterize the perinatal period, with an emphasis on the understanding of the male gender perspective. The exploration of PAMA psychometric properties further highlights the need to adopt a gender sensitive approach in the development of screening tools, with the general aim of improving the quality of assessment of perinatal mental health in both parents. In fact, the findings of the present study indicate that gender sensitive screening tools might be especially useful for the detection of perinatal affective disorders. Further validations of both PAMA and PAPA in other countries are recommended to support the advancement of research and clinical guidelines for the appropriate screening of both expectant parents’ mental health.

## Figures and Tables

**Table 1:** The Perinatal Assessment of Maternal Affectivity (PAMA).

Item	Italian Version	English Version
1. Anxiety	Tesa, ansiosa o preoccupata	Tense, anxious or worried
2. Depression	Triste, giù di morale o depressa	Sad, down, upset or depressed
3. Stress	Sotto pressione, stressata	Under pressure, stressed
4. Anger	Irritabile, arrabbiata o polemica con gli altri	Irritable, angry, or had arguments with others
5. Interpersonal	Ho avuto più del solito difficoltà nel rapporto con gli altri o altri hanno avuto difficoltà nel rapporto con me (il mio compagno, familiare, amici, sul lavoro, ecc.)	I’ve had difficulties getting on well with others, or others have had difficulties getting on well with me more than usual (e.g., my partner, family members, in-laws, friends, at work, etc.)
6. Somatization	Fisicamente male (mal di testa, dolori muscolari o articolari, problemi digestivi, gastrointestinali, cardiologici o di pressione, disturbi urinari, ecc.) (anche uno solo di questi)	Physically unwell (e.g., headaches, muscular or joint pains, digestive, gastrointestinal, heart or blood pressure problems, urinary disorders, etc.) (one or more of these)
7. Sleep, Eating and Sexual Desire	Ho avuto problemi a dormire, nel mangiare o nel desiderio sessuale (anche uno solo di questi)	I have had some problems with sleeping, eating or sexual desire
8. Addiction and Risky Behaviors	Ho sentito più del solito il bisogno di fumare, bere alcolici, assumere droghe, giocare d’azzardo o utilizzare internet, oppure dedicarmi ad attività pericolose (guidare a velocità elevata, praticare sport rischiosi, mettermi inutilmente in pericolo sul lavoro, ecc.) (anche uno solo di questi comportamenti)	I have felt the need to smoke, drink alcohol, use drugs, gamble or use the internet more than usual; or felt the need to take risks (e.g., driving very fast, doing dangerous sports, unnecessary risks at work, etc.) (one or more of these)

**Table 2:** Characteristics of the study sample by parent gender.

Variable	Categories	Mothers (*n* = 225)	Fathers (*n* = 215)
Nationality	Italian	87.9% (197)	74.3% (156)
	Foreign	12.1% (27)	25.7% (54)
Residence	Northern Italy	91.7% (199)	92.3% (191)
	Central–Southern	8.3% (18)	7.7% (16)
Education	Primary school	0.0% (0)	1.0% (2)
	1st degree	10.5% (23)	15.6% (32)
	2nd degree	48.9% (107)	56.1% (115)
	University	40.6% (89)	27.3% (56)
Job	Unemployed	18.7% (37)	14.0% (30)
	Employed	81.3% (161)	86.0% (185)
Marital status	Single	7.5% (16)	6.5% (13)
	Separate	0.9% (2)	2.5% (5)
	Cohabitee	38.5% (82)	38.7% (77)
	Married	53.1% (113)	52.3% (104)
Number of children	Primiparous	41.4% (91)	50.2% (108)
	1 or more	58.6% (129)	49.8% (107)

**Table 3:** Maternal scores on PAMA by group.

Other Children *		Nationality		Stressful Events	
None (*n* = 91)	One or more (*n* = 129)	Italian (*n* = 197)	Other (*n* = 27)	None (*n* = 159)	One or more (*n* = 66)
0.87 (0.51)	0.95 (0.51)	0.95 (0.51)	0.70 (0.41)	0.89 (0.52)	0.99 (0.49)

Data are expressed as means and standard deviation (in brackets). Total score range 0–3. * *p* < 0.05.

**Table 4:** Descriptive statistics and differences between maternal and paternal scores on PAMA/PAPA.

Item	Mothers (*n* = 215)	Fathers (*n* = 215)	*z*	Effect Size of Differences (*r*)
1. Anxiety	1.20 (0.77)	0.99 (0.77)	−3.41 **	−0.16
2. Depression	0.46 (0.66)	0.23 (0.48)	−4.78 **	−0.23
3. Stress	0.89 (0.80)	0.86 (0.79)	−0.45	−0.02
4. Anger	0.92 (0.86)	0.72 (0.82)	−2.82 *	−0.14
5. Interpersonal	0.52 (0.66)	0.41 (0.64)	−1.90	−0.09
6. Somatization	1.14 (0.85)	0.54 (0.71)	−6.87 **	−0.33
7. Sleep, Eating and Sexual Desire	1.31 (0.85)	0.58 (0.69)	−8.58 **	−0.41
8. Addiction and Risky Behaviors	0.08 (0.35)	0.23 (0.52)	−3.45 **	−0.17
Total PAMA/PAPA	0.92 (0.52)	0.55 (0.41)	−9.00 **	−0.43

Data are expressed as means and standard deviation (in brackets). *z* = Wilcoxon signed-rank test; * *p* < 0.01. ** *p* < 0.001. Item 8 was not considered in total mean score for mothers, coherent with the final PAMA version derived from CFA.

## Data Availability

The data presented in this study are available on request from the corresponding author. The data are not publicly available due to privacy and ethical reasons.
